# Endoscopic mucosal resection with a dedicated bipolar soft snare for large flat colonic polyps

**DOI:** 10.1055/a-2158-7895

**Published:** 2023-09-15

**Authors:** Shunsuke Yamamoto, Miho Kozuki, Kensuke Matsushima, Yuko Sakakibara, Ryotaro Sakamori, Eiji Mita

**Affiliations:** Department of Gastroenterology and Hepatology, National Hospital Organization Osaka National Hospital, Osaka, Japan

When removing large flat colonic polyps (LFCPs) en bloc endoscopic submucosal dissection (ESD) may be suitable; however, it requires significant expertise and a long procedural time. The use of a conventional monopolar snare for endoscopic mucosal resection (EMR) of large lesions increases the risk of deep mural injury 1 . Bipolar snares are safer than monopolar snares because the current passes only through the tissue between the two electrodes, so causing minimal damage to the muscle layer 2 .

The Dragonare dedicated bipolar soft snare (Zeon Medical Inc., Japan) has a unique mechanism where one electrode is at the tip of the snare and the other at the sheath, which allows uniform cauterization of the tissue. The soft nature of the snare allows stabilization of its tip at the distal margin of the lesion while opening the snare, thereby enabling en bloc resection of LFCPs. This requires a slower cutting technique compared with monopolar devices. Herein, we report two cases in which EMR was performed using the bipolar soft snare (26 mm) for LFCPs ( Video 1 ).

**Video 1** Two cases in which endoscopic mucosal resection is performed using a dedicated bipolar soft snare (26 mm) for large flat colonic polyps.


The first patient was a 78-year-old man who had a 25-mm nongranular laterally spreading tumor (LST) found in his descending colon ( Fig. 1 ). Although submucosal invasion was suspected, the patient wanted to undergo endoscopic treatment. Underwater bipolar EMR (BEMR) was performed and the lesion was completely removed, without the occurrence of any adverse events ( Fig. 2 ). Pathological examination confirmed the lesion was a submucosal invasive adenocarcinoma ( Fig. 3 ); however, the patient chose not to undergo an additional surgical resection.

**Fig. 1:**
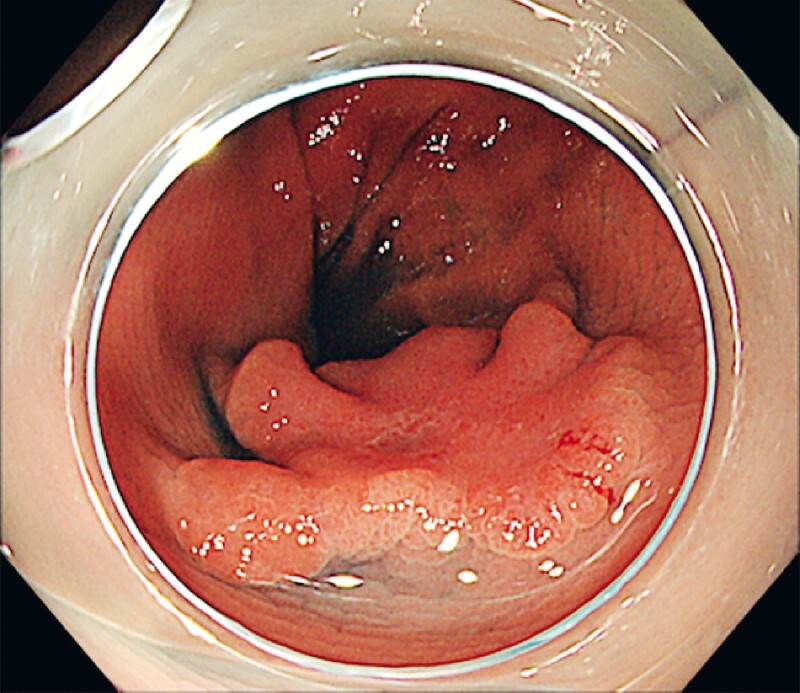
Endoscopic image showing a 25-mm nongranular-type laterally spreading tumor (LST) in the descending colon.

**Fig. 2:**
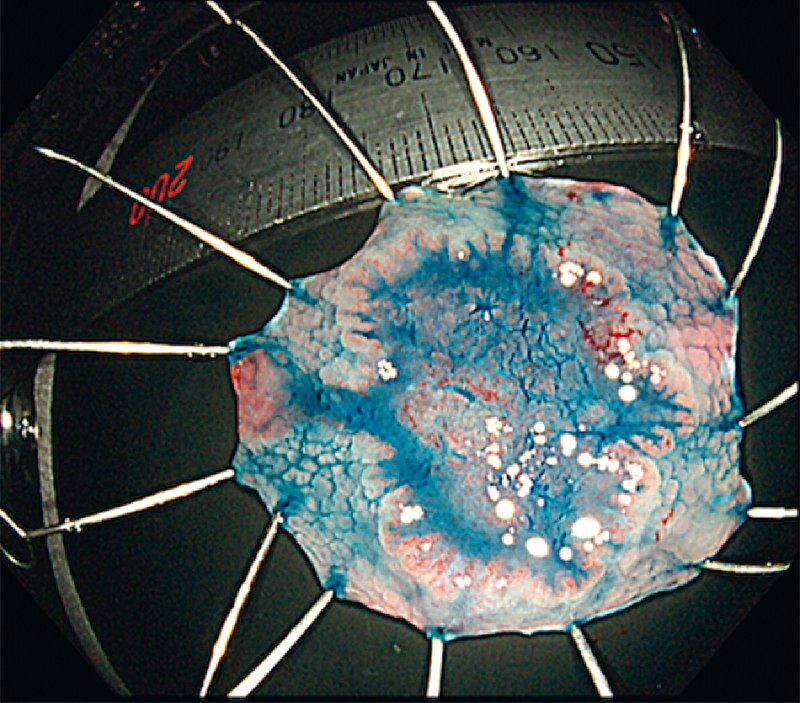
Macroscopic appearance of the specimen obtained by underwater bipolar endoscopic mucosal resection.

**Fig. 3:**
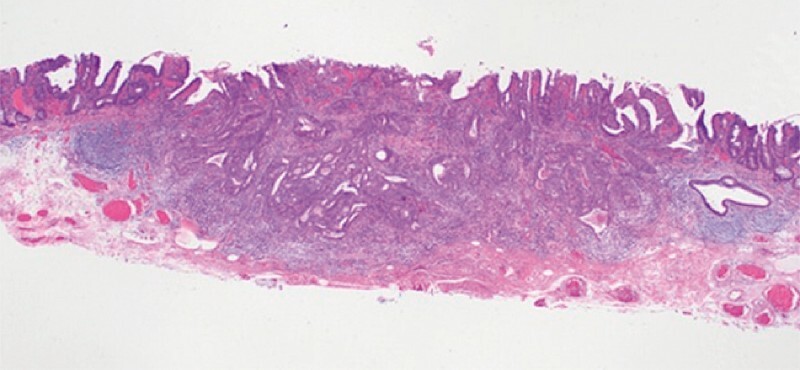
Microscopic appearance showing submucosal invasion of the tumor.

The second patient was an 86-year-old man who had a 30-mm granular-type LST found in his ascending colon ( Fig. 4 a ). Injection-based BEMR was performed, and the lesion was completely removed, without the occurrence of any adverse events ( Fig. 4 b ). Pathological examination revealed a focally high grade tubulovillous adenoma.

**Fig. 4:**
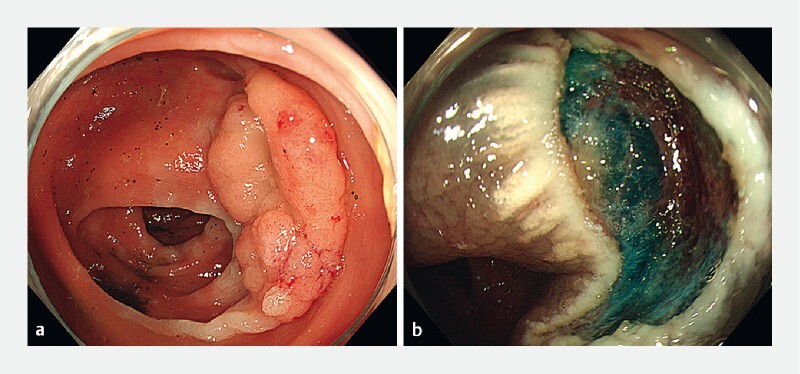
Endoscopic images showing: **a** a 30-mm granular-type laterally spreading tumor (LST) in the ascending colon; **b** the appearance following injection-based bipolar endoscopic mucosal resection.

BEMR with dedicated snares could be a new alternative when treating LFCPs. ESD can be replaced with BEMR for eligible polyps.

Endoscopy_UCTN_Code_TTT_1AQ_2AD

## Notes

Endoscopy E-Videos

https://eref.thieme.de/e-videos
*E-Videos* is an open access online section of the journal *Endoscopy* , reporting on interesting cases and new techniques in gastroenterological endoscopy. All papers include a high-quality video and are published with a Creative Commons CC-BY license. Endoscopy E-Videos qualify for HINARI discounts and waivers and eligibility is automatically checked during the submission process. We grant 100% waivers to articles whose corresponding authors are based in Group A countries and 50% waivers to those who are based in Group B countries as classified by Research4Life (see: https://www.research4life.org/access/eligibility/ ). This section has its own submission website at https://mc.manuscriptcentral.com/e-videos
